# Supplementary material for: Integrated Analysis of mRNA and microRNA Elucidates the Regulation of Glycyrrhizic Acid Biosynthesis in Glycyrrhiza uralensis Fisch
Source: Int J Mol Sci. 2020 Apr 28;21(9):3101. doi: 10.3390/ijms21093101 (PMC7247157; doi:10.3390/ijms21093101)
Supplement: Supplementary file 1 [file ijms-21-03101-s001.zip › Supplemental Tables/Supplemental Tables/Table S19. Promoter regions sequence of structural gene sequence..pdf]

Table S19. Promoter regions sequence of structural gene sequence.

Note: TAACTG is a R2R3-MYB binding domain, marked in red. ATG stands for start codon marked in yellow.

>Glyur000218s00011642.3 gene=Glyur000218s00011642 AACT

AATCGATCTAAGTCCTCTAAAGTGAATTAGCCGAATCCTTTATGAATAACCACGATTGTT
TAACTGATTACAACTTCATTACTTTTTTTTACCMCAAAGTATTTTTGTCCAATTTCCGTT
GTCTACTTCTTGGATTGGACCTCACCTATGGTTCTACGCACAAGGTTTTAGTTGATTAGT
AGGTGGAGCAGAGTCTGCACGTGTGACTTCATAAGTTTATATGAAAAAATAAAAAATAA
TTAGTGTGCWTCGCATGCAAAGCAAGGCATCCCCATCCTATATTTTAATTACGCCATTTA
TTTCTAGACCAATTACCCCACCACAAATCAGTTGCCGAAGCTTGACAATTACCAATTCA
TTGTTGTGCATAAAATTAACAAAAATTTAGGGAAAAAAATGTGCCCAAAACACCATCTA
TCAATTTTTAAGCTAACGGAATTGTATTTCTAAAAAAAAAAGAAAAAGGGCTAACGAG
TAACGGGATTGCCCATGCCCTTGCTCGGTCGGCACTGTTATAGTCTAGTCCCTAAATATT
TGATTATTTATCATTTTGTATTTTAATTCTTAATTAATGCAATGATATGAGTTTATTCCCTTA
AAAAACATTATTATCAACATTATAGCATACATATAACTAATAAATTAATATTCTCTCTCAG
CAGAAAAATGTACAAGGTTAAGTGGTTAACTATATAACTAGTTGGAAAACAAGAGAAG
TGGTTGGTGGTTTTGGCCTCAACTCAAYACGGCATCACAGTGAATAGTAGTTCCGTTGC
ATTGGGGAAGGGTGCGTGTACCTCGTGGCTCGTCCCACCTGGCACTATATAAACAGTC
GGATCAACCAGACATGCTTCTGCACTCGTCACACTTCCCTCCCTCGTTTTCTCACTCGC
TTCCTGCTCCTTAACCTCTTCAACAACAACACCACCTCTTTACCTTAAAGGTAACAATC
CTTCCTTCTCGTACGCTTCTTAGCTTAGCTCAACCGTTTTAGATCTGTGTTTCGCTTCAT
AAGAGGACCTGAAATGTCATGTTTGATGCATTGATTGTTATCTGTTTGARGATTTGCTAT
TCCTATTTCTCTGCTAGATCTTTATTTATTCTTCCCTCGTTTCTCTGATCGTTARTAGTTCG
TCCCATTCATCTTGTTTGCTTACATCATTACATGYCAAAATTTAGGCAACTCGTAGTGAG
CATACAGTGTTGCGTGATTTTCTCTAATAATAAATATGTGAGAAAGAACGGAAGGGGAA
GAAACATAAAATCTCGAGCTGATTTTTTTTTCAAAAAAAAAATTCTGAATGGTTAATTA
TGTAATGATCAAATTGCGCTACCATTTTTGTATTATGCAACATATGTTATGGCTTTGAGTT
GAGGTAGGTGGAATGTGTAGTTAATGGAAAATAGTTGATAGTCATGATTTTTTTATAAAT
GGATTTTTTAGTTGAAAGGATTAGGGATCTTCACCAGTTTGACTTATCAAACAACCTTG
TTTGAATCTTTACAAATATTAGTAGATTTCTATCAAAATTTTCTGGCTGCATAGCATTCCT
AGCAGTTGTTTGGAATAAGCATCTTCAGTTCTTCTCTGGTTCATAATTCAACAAATTTAC
AATATTTCAATTCTTCTCTGTTGAATTTCTTAACTACGTAATTTTTGTTTTGGTTCACCTC
AATAGTAAGATGGCAAAAAAGGAAAGGAAATCGGGGAAAAATAATATAGACAAAACA
AGGTTGTGTTAGTGTTGTTACACTTCTAAGAAATTTGTCATTTTCTCACATTTGTTCCTA
TCATTTTCATCAAGAAATCGAAATCGGTCTAATGCAATATTATGTGGAGTAAGACAAAA
ACACCTGAATCTTAAATGCATGCTCATACAAATACAATCAATCATATATGAAGCTAGTTC
TTATAGATTATAGATGTGTTAGTTTGTTAATTTAATTTACATACATCCATATATTATGTTTTG
GTTACAGATTCCATTCTCATCTATCAATG

>Glyur000682s00024324.1 gene=Glyur000682s00024324 HMGR

CACTTTTGATCCCTAGTTATCGCGAATATGTGAGTTAGGTCCCTAGCTTTCAATTGAGCC
AATTGAGTTTCTAGTTTTTCAATTGTTGCAATTAGRACTCCAGTTTTCCAATTCTTGCAA
TCAAGTCACTCTATCGATATGCCGTCCAATTTTTAACACTTAGAACCTACAAGGTACAC
GCGGGACTGATTTGACACTGAATTAGGTCACACTAGCACAATGTAGGTTGCCAATTTTT
TTTTCCAAAACAAGTCGGTGACACGCATAATGCAAAACATGTTTGTCATGCCATTAGTA

CCAATGTGATCCAACTCAGTGTCAAATTAGTATCACGTAGGTTCTGGGTGTTAAAAATT
GGATGACTAGTGACAGGGGACCTGATTGCAACAATTGAAATACTGGCGAATCGGCTCA
ATTGAAAACTGGAAGACCAAAATTGCACAATCACAATAACTGAGTTATCAAAAATACA
ATTATACCTATTACTTTTTTAACGTCTAGTTTACCTCCTAATAATTGCTACCACTTAAGTTT
GTACTAAATTCAGTAAAAAAAAAAGTTTGTATGAAATATAATTGATTAAATTTTTTGATAT
AACCATATAATGATACGAGGGATATAATAGATAGGCACCTCGCCAGGATTCATGATCACA
AGATTATTTTTTTTTTTGTTTCTAAAATCACAAGATTAATTTTTTTTGTTTCTAAAATCA
CAAGATTAAATAATAAATCAAGTTGGCATTGATTTTAAAATAAATAATTAGATGTAATGT
GTTCATAGGCATTGTAATATATTCTTAATTAAAAAGTATTTAATAAGAAAACTACTAACG
GCACATTTTCTTCAACATGTGATCTTTTTATAATCGGTCTATTATTGAAAAAAATAATATA
TTTTTTTATAAAAAAAAGTCATGACAGATTTTGGACTACTAATTTGAAGGCATATTCACC
TAATTTCATTGTATAAAAATGACTTTCTCTCAATGTTTGTCTTTTAGGTATGTGCTCATGT
GTATTTTGTGTGAATTTAATTGATCAAACTGTTAAATTAAAAATTATAAATACATCAAATT
TAAAAATTATTTAATTATTCACTTTATTTTATTTTAATGTATGATATATTATTTAAGATATATA
AGTACATATCTACTATTAAAATATACATCTATAAATATCTAAACATACATAATCAAAATAAT
ATAATATGATCTAATGATTAAGAAATTATTAAAAGTATAATATACTACATATAAAATGTTCA
AAGAGAGATACACCTGTGTGCGACGTCCATTTGATGACAAATCATAACTTACATATATTT
TGGAAACTGTTTCTCTATCTTCTTTTTTTTATGAAATTAATTTCTTTATACGTGCATGTCC
AACTTTCTCCAATATATTTTATTTATCATGTTCAAAGAGATATGATAAAATTTAACCAAAA
AAAAAATGATAAAACAAAATAATTTTTTACAAATACACCCTTATTTTATTACGTCCACAA
AAAACGCACGTGGAGAGAGACACCACCACCATGCAGATAAAAAAGAAATGCACGTGG
AGAGTGATACRAATATGAAATTTCTGCCGTTCCAAACAAAATAAAAAAGGGTTAAAATA
AAATATAAAATAATAATAATAAAAAAGGAAGAAGCGCGAAGGAGGATTGCACGCTGTC
CGCGTGGGCATCAAACAAAAGAAGAAAAATGAACAGCGCGCCAACCGCTGTTTATTT
WTTTTTTCTTCTTCTTTTCTTTCTTTCCTATAAAAAACCTTCCAAAGTCTGTCATTTGCT
TTCAGTTCCAACAGTTACCAAAAATTTCCCCTTTATTATTATTATTATTATTTCTCCCTCCC
ACATTTTCCTCCCTCTCCTTTTCTTTTCCCTCCCCCAAACAAACACCCAAACAAAAACC
CTTCTCAAATCCCTCGCTCCACTTGACTCGCTTCTGGGTTTTGTCCATTTTCCTTCGTAC
CCCCTTCYCTCGTCGGAAAATG

>Glyur000069s00004081.1 gene=Glyur000069s00004081 MK

AGCCAGAGTTTGGACATATATAGATATACTCCTAATTATGATTCTTTGTTTGATTATGCAA
CAGCAAATACACGGTTGGATTAGAGTTAAAACCAAAATTGCACACTTGAAGTACTTCAT
AACTGGCACCCCTCAAGGATGAGRACAAAATCCAATAAGGTAAGTAAGAAATTAAACG
TGAATGACTAATCACTAATGAGTCATTCATGAAATCCATGCATATATTAGAGGTATACAC
AAGGAGAAGCAATCATTACCAAAGTGAAATGCACAATATAAAGAAGAGATTAGATTAG
TTTGTTTCTCTCATATCATTTCATTTCATTTCATTTTATTACATTAACTTGCTAGAGAAAAC
GTTGGCATATGGCTAATGTCACAGAATGTAGTGATCCGTGGCCATTTAGAATCAAGCTT
ACAACTGATTGCATGCGTTGGGATAGCCTCAAAATCTCACCTTGCAAGGAGTTTGAAG
AGCACATTGTGGGACACATGAATGAGAGTAGCCGTAAGGCTTTGGAGTCTTGGATTCC
GGTAAACATATTGATTTATGATGTTGATACTTGTGAGACTTATGAGGCTAAGTTGTCAAA
GAAAGAGTCATTTTGGTTTGATCCTCTGCCTGTTTTGGGTGAAAAACCAAAGATGGGT
GCTGGTGAATATTGCTCTTCTTCTAAGGCTGTTGTGAGGGAGCCACCTTGTTATGATTTG
GAGAAGGCAAGGGAAGAGTTTGCTTATTCAGTTGAGCCTTTTAGACACATTATAAGGA
AAAGGGATCTTAAATATGATCAAGAAATTGGACTTCGCTTTTCTGGTGGCAAAGTCATA

GTAGGATTTGAGTTTTCGGTTCTGCATTCTTCTTCTTCTTCTTCATTGTTGGATATTC
ATTGCTTCAGAGCCTAGATTGTGAGTTTATAAATAAGGTAAGAAAGGTAGATGAATGTT
TGTTTTGCATTTTATATGATCAATCAAGGAGTTGTAAAATGTTGCATTTTCAATAGTGGT
TTTTGGACTTGGGAGCTACATAGGAAAAAAGATAGGGCACTTTTGTCTGAATGTTTTTA
TACTTTTTGTCTTGATATATATTCAAAAGTCATATTGAATATGACTAATTTAAGTTAAGTT
GATTAAGCTCCAGCATCTGTTTTTTTTTTCATTAGCAAAAACTCAAACTTGAGACCATG
GTTAGGGAAAACCAAACTCTAGTCACTTAAATCTAGTACTAGGTATTAAAAAAGGTTTA
AATATATATTGAGTTCAATATGTACCTAACACCCTTATGTCTTGTTTGATTTTATTAAAAA
AATTGAATTCATTATTTGTTGTTACTATAGTAATACTATAAGATATACATGTAATTATTGTG
AATTATAGGCTAAATAAAAAATATTTGAAGTTTAGCATTCATAATTTACAACGTCAGGTT
AGAGAGTTGAGATTGAATTAAAAATAATTAAAAACAGAAAATAAATTAAAATTGATTTT
TTATATGAAAGGGAAATTAAATAAAAATTAAAATGAGGATCACGTTTGGGGAAAAAAA
AAAAGAAGAGAAATAAAGGCAATAATAAAAAAAAAGGCACGCATTCCACATGGAGAT
GGAGATGGAGCGGGACTGGACTGAGTGGAGTGAAACAGAAGACAAGAGTACGACAC
ATATCAGAATCAGATGACAATGCAATGAGTTGAGTTGAAGAGATGAATGGGAGAAGAA
AGTGAAAGAGCTTATATGATCAAGATGCATGTATTTTTTTTTTATATTTGAGACCAGAAG
GAGTACATTACATAGCTACTTAGAAGAGAGAGAGAGAGATTATTCAGAACAGAAAGAT
GCATGTATTTTTTTTTTATATTTGAGACCAAAAGGAGTACATTGCATAGCTACATAGAGA
GAGAGAGAGAGAGAGAGAGAGAGAGAGATTATTCAGAACAGAAAGACACAGAGACA
CTGATAGATAAATAGATAGATCCATCAAATCAAATCAAATCAAATTCAAATG

>Glyur000002s00000233.1 gene=Glyur000002s00000233 MPD

GATTTGGCACAGTTGCCTGCTCACTCATACCTTATTTGGCTATACCTTTTGCTCTGTTTTC
ATTTTGACTTGTGACATATAATTAGGGTTGAGTTGTTTTCTCAATTTCTAACTTAGAATTA
TTACATCAAACACTCTGCTTATTTATTTATATTATACATTTCAATTATTTTTTTACTGTGCA
TCTGAATCTTACAATTTGACACGATTTATGATTCAAAAAATTAGCTCAGTGATATACAAT
TTTAACTGCAATTTGATAACAATGGTCATTCACATGTTAAGATGACTTCGCGAGAAATA
AAATGACCCCTTTGTCAACAATATGAGTGTAAGTAATGCCGGGTTGAATTCAAGACATG
TGAAATGATATATTCTGCCTGTTGCTTTGCATAATAATGCTCAGATGGATGGAGTTTGCC
AATTATAGGCAATAGTTCAACATCTAACAAATACCTGCACAGGTTTAGATGAAAACCCA
AAAATGAATGATAGTATACTATAAGGGAACTACCATAATGATTCAAAGAGAGTAGATTA
ATGATGAAAAACAAAAAAGGTAAAAACCTGTCACCATCTTTGTCAAGCTGAGCAAAC
AACTTTCTAGCTGGAGTATCTATTGAATTATCGCAATGATGTGAATCATTGTGACTTTCT
GCATCATAGTTTCTTACCAAATCAAAGAACCCATGGAATTTTTTTTTAAAGTTGACGTTG
CCATCTCTGTCTGTATCTCTTTCGCTATAAAGAAATAAAAGGATAAATCAAGCCACAGT
GACATAAATAAGCTACTTGTATACAAACTAAAAATTTACCATTCACTTGATAAAAATAAA
ATTAACTACAAATATTATCCACATATCTAATATTTCGCACAAGTCCCCAAAAAACTTATTC
CGATATTGTAACTTCTTCTAGAGGATGTAAATTGAATGCAAGACCAAATTCATTTTCAAG
AACAAACCAACCATGCATCCATCCCTACAAGAAAAACAATTTTTTGCGGCAGTTCAAA
CCGCCGCTATATTCAATTTTGCTGCAGTTAGGCAAGGGCTGCAGTGTCAGGCGTCGGCA
ATCTAATTTGCGTCGGTTACAAACAGCTGCAATCATCTAATATTTTAAAAAAGATTAAGC
GGCTCGGCGAGAAGTTCCAGTTAACTTTTTTAAAAAAATTCAGCGGTTTGTTTACTATC
ACAAGATTTCCTGAATAATCTTTTATTAATAATAACAAAATAATAATTTTAAAATATAAAA
TAAATATTAGAAAGGACAAAATATTTAAAATAAAAACACTATTCCATAATCCAAATACAT
TCGAGTTTGCTCTTGGACTTGCTATATCAGAAAGTAAAAAAGTTTTGAACTTACAAGAT

CCAAATAGCATGAATTATATAGAAGAACATACANNNTCAATTAGTATTGTTTTTTATATTA
CTCAATTAAAATACACTTATCAAATTTTATTTGTCGGAAAAAATATAAAAAAATACATTAG
ACTTAATTTTGTGTGTTTATTAAGAGCTGAGAAGAAAAAGAAAAACAAGAAAGAAGG
TTGGTGATGGCGACACAATTCAAATCGAAATTAATTAACCCCAAGCATGGGTTTGGCAG
GGTATAACGGGGAGTGACCCTCTGAATTTGGAACCCAAAAAAAGGTGTTTTGATTTAA
TTAAGMCTCAAAGAGATTTGCACCCCTTCTTCTAATACCTTATGCTATATARTAGGCGTC
TTCTTCCTCTGTAATTCCGTTGCTATAACATAACATTCAGATCACAGACAGGGATCAGAT
CCCTTTCTCAACTCTTAATTACTGCTAATACCCATTTCTTCACTTCCCATTTTCTTCCCTT
CCTTTTTTCCCGCTGTTTTCTGCGGGATTTGAAATTCCCGGGGAAAACCCTGATTCCCC
TGATTCAGATTTTCCTTTTGCAATCAGTGTGTGATTTGTCCTTTTGGTTGAATTCGATTG
TTTGGTTGGTTGGTTTTTTCGGGGTGCTGGAAATG

>Glyur000089s00008825.1 gene=Glyur000089s00008825 SQS1

ATTTTTACAGAGACCCAATTCATATTAAAAAAAAAAAAAATGTAAGAAACAGTTGTCAT
TTTAGCATTTTTTCATTTTCAATCGCCATGTAGGGTTATAAATCACTTTTATTGGTCAAAT
CTCGTTTTGTACTACCTCTAGTCTAAATCTTTAGTGGTTTTAGGTTTTTATTTTTGTCTCA
AAACTTTGGTTGTTTTAAGATTTTAAGAGATAATTTATCACATTTTTTCAACTATACCCTT
AATATAATTAATAAATTTTGAGTAGAGAAATAATTATGATAGTTGAGATAAATAAATAGGA
TTACTTTAGTTTAAAATTTATTCAATTAATTATTTCTTAATTTATGTGTCAAAACCTTAAAC
AACAAAAGATTTGGATCGGAGGGAGTAGTTGTTTTTGTATTAAAAATTTCATAATAAAT
AGTACGGCTTAAAATAATTTTGATGGTTTAAACCAGTGTAGTTAAAACCGGACCGGTTG
AACCGGGAACCGGACCCCGGTCCGGTCCGACACACCCTGAGAATCGCCCTACGCAAG
AACCAGATAAAACCGAGAAAACAAGCTAAAAACCAGTTGGACCGGTTCAATGATTTA
AAAAAATCCAAAATTTCAATTTTCAATTTTTTTATTTTTTTAAAAGAAATAGATATCACTT
TCTTCAATTATTTGTAATTTGAATATTTTATGTTATGAATTATGACATCTAGTAGTAAAGTT
TAATTTTTCTTTATAAAGTTATGACTTTTGTGTTAATTTTTACTCAATATTGTATAGGGTTT
AGGGTTTAAAATAATAATATTTAATTATATCCCAGTTCAACCGGTTGAACCCCGGTCAAA
CCTCTGAACCTTGAACTCCTGCCCTCATCGGTTCAATGACCGGTCCGATTCTGACTACA
TTGGTTTAAACACAAATTCTCTAATCAATGATATTGAATGCAAATGGTTAATATAATTCAT
CTGAAAGACGGACTAAATTGGTACTTGTATTTTATCGATGAAATAATCTATAATAAATTTT
TTTGACTTATTTTCAATCAAATACATATTTCCTCTGTTCCTTTTTATTTGTTGGTTTTTAAG
AAATTTTTTATTATTTTTATTTGTCGTTTTGATAGTTTAAGGTTACATTTTGATAATTATAC
CCTTAACTCTATTAATTGTTTACATATTTTTCATAAATTAATTAATGTAGTAATTGAGAAGT
TCAAAGATTAATTAAATATAATACATCTTCTCCCAAAAATTATTTTACTTTCTAACAACAT
AAATTTATTACCAGTATACATGTGAATGTAACTGTATCTAAGACTAATTGGATAATTAAAT
AAGGGATTAATAATGATTAAAATGAAGGGACACATTAATTGATTTATCTATTCTCAAAAA
TTAATTAAATAAAAATATAATAGAAATTTTTTATTAATTAATTAATTCTTTAACTATAATAA
AATAATCTAAAATGACATATAAAAAAGAACGAATGGAGTATTAGTTTATGACTCCTATAT
TGTACTATAGTGGGTTAAAAAAAAGGCAAATTCCCTCCCACGGGAGGGAACAAGGAG
CCTGTCCGTGTAGAGCAGGGTGACTCCCTATAACTAGACGTAGCAACTAGAAACTAGC
AAGGAAAAACCAGAGACACGTATATATACACATTTATTGATTGCGAGTGCACGTTTCTA
GCAAGGCACTCGCGTCTCGCGTTGCCTGCATAAACAAAACAAACCAGCCCCACCAAA
CCAAAGGGGAAAAATAAGAAAAAAACGACAAATCTCGAACGGTAATTATTATGCTGCC
AGTGCCTGCCAACATTGCGAAGTACTACTAGTAGTCTAGTACTGGTACTTAACCCAACC
CAACCCACCGCGTGCATTGCTTGATTCGGACACACAAACACAGCACAGCACAGCACA

CCCGCCCACGAGTTCCCTCCCGGTTTTCTTCCATTTTGCATTTCAATTCTGAGATTTCGA
TTTGGATATCGCACTCGGAACTATG

>Glyur000561s00023451.1 gene=Glyur000561s00023451 CYP88D6

TTTCACTTTCTCTCCTCCCACCTAATTAATGCATTATCTCTATCTCCATTCATCACAGAGA
GGAGAGAGAGCATAAATAAATAAGGGTAACTTTGGTAAAAAAAATAATTAATACATGAC
ACATTTTCAAAAGTCTCTTATAAAAAAGAGACAAACAAACATGCCAAAAATATCTTATA
ATTAGTTACAGATGTAGTATATAACAAAAGAGAAATGTTATATCATCTTATTACTTTATAA
AGTAAGTATTTAATAAAAGAATAATCTAAATCCTCCTATTTTTTCTTCCTACAAATCCTCC
TAATTACTAAATAGAGAAAGTATTAAATATTTTTTATGTTTTCAATATATTAGTTATATTCT
CTTTTTACTTTATAAAGTAGTAAGATAATATGTAGAAGGATTTGTAGGAAGATATAACAT
TTCTCTTAATAAAAAAATTGAAGCTTAAAAACATAAAAAATATTTAATATTTTTTTATATAA
TAATTAAGAAAATTTGTTGAAATAAAAAGTAAAAAGATTTAGGATTATTAAAAAAAAAT
GAAAAATGGGATTGCATTGATTGCACACGTACTTGCAGCTCACTTTATTATGGCAAAAG
ACTTGCTACTAGAAAAAAGGTGGAATCGGATTCTCTACGGGGCTTTTTTATCCCATTAG
CACACTTTTATTTATTTATCAAAATAGTACACTTTTGAATATAATATTTATTTAAGTATTTT
TTATAAATTAAATATTAATAGTTGGTTTAAGATTATAATTAAGTTATTAATTAAATTAATATT
TAATTGTTAAATATTTATTAACTCGATTTGTCAACTAATTAATAATATAATTATAGAAAATA
GCTCAAATTGATTCAAAATTTAAAATATAAATATAAAAGTGTATAAACAGAAATGATAAA
ACAGTGAACTAAATGCAAATGATAGAAATTTTCTCAGTGCTGCGTCTCAGCTCCTGAAC
GGTAAGGGCAGTTTTTCTTGCTATGCCCTTCATTTCGATACAGTCCACATCTCTTGGGAT
GACGACTCTTTTCCATTTGATCCATTTCTGTACGAGTTCTCGTCGACTGTGGACTAAAA
CAGTGAACTAAATGCGAATGATTGAAATTTTCTTAGTGTTGCATCTTAGCTCCTGAACG
GTAAAGGCAATTTTTCTTAGTATGTCCTTCATTTCGACACAGTCCACATCTCTTGGGATG
ACGACTCCTTTCCATTCTATCATTTGCATTTTGTTCACTATTTTATCATTTCTGTTTGTATC
ACTTTTGTATTTGTATTTTTAAATTTTGAATCAATTTGAGCTATTTCTATAATTATATTATTA
ATTAGTTGACAAATCAGGTTAATAAATATTTAACAATTAAATATTAATTTAATTAACAACT
TAATCACAATCTTAAACCAACCATTAATATTTAATTCATTAAAAAATACTTAAATAAATAT
TATATTCAAAAGTGTGCTATTTTAGTAAATAAATAAAAAGGTGTTATTTTGGTAAATGAA
AAAAAAAGGTATTATTTTGGTAAATAAAAAAAAAGTATGCTGTAAAAAAGCCTCTCTAT
GGCGGTGATTGCGGAAACAAATCAACTTTTTCAGTTGACACGACAGTATTAACTGTAG
ATTTTTTTTTTTGGTGAGTCTATTATAACTGCAGATTTACACCTTATAGTTTATTCAAGA
TCTATCTAGGCCCTATTTGGTATAACAGTTTATTCTACAGTTTATGGCATAAACTGTATAA
ACTATGTTTATAATTGAAGAAAAAATATGGTGAAATTTATTTCATATAAGCTATTTTGATA
AGCTATCCTGGGTAACTTATGAAAAATAAGCCCAAAACAGCTTATGAATCAATCATAAG
CTGTGTTCATAAGCTCTGCATAAGTCAATCCAAACATGCCCTGATCTTTCTCATTTACAT
CATGAGCAATCAATCAACTCTGTATATAAAGAGAGGGATCCGATGAACAACGAAACAG
ATTCAGAAAAATG
