# Supplementary figures and images for: Integrated Analysis of mRNA and microRNA Elucidates the Regulation of Glycyrrhizic Acid Biosynthesis in Glycyrrhiza uralensis Fisch
Source: Int J Mol Sci. 2020 Apr 28;21(9):3101. doi: 10.3390/ijms21093101 (PMC7247157; doi:10.3390/ijms21093101)

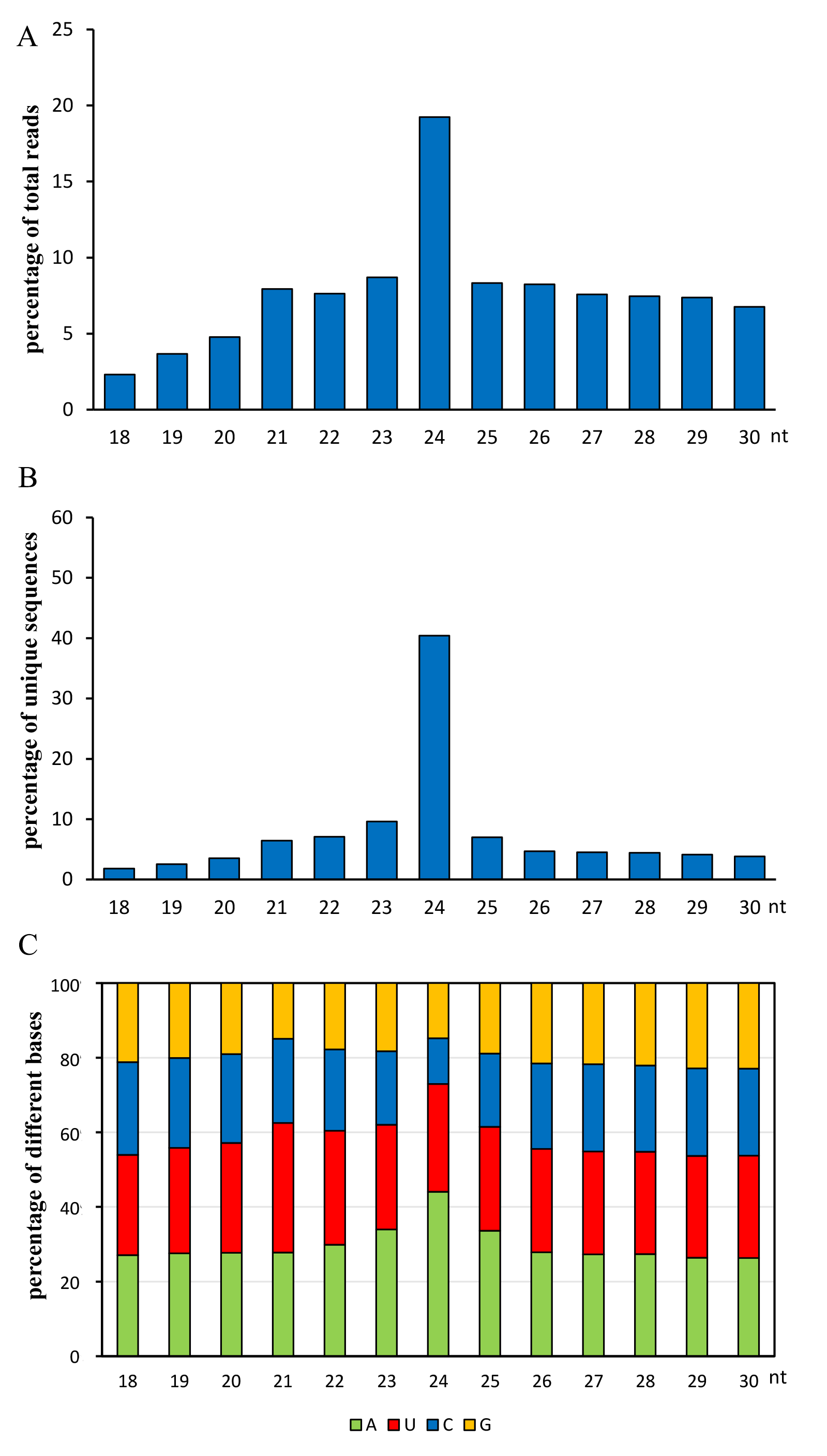

Supplement: Supplementary file 1 [file ijms-21-03101-s001.zip › Supplemental Figures/Figure S1. Populations of sRNAs in G. uralensis..tif]

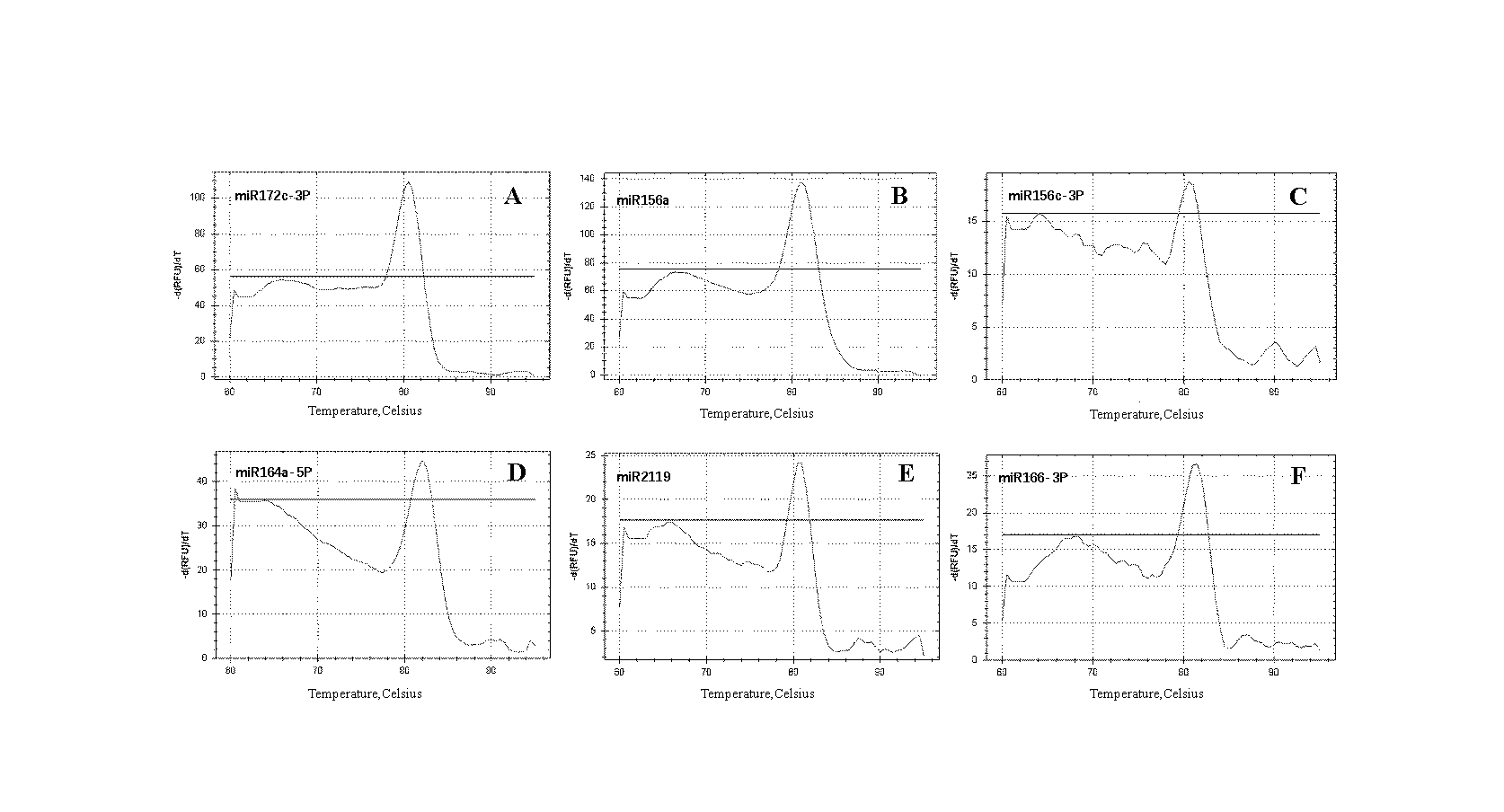

Supplement: Supplementary file 1 [file ijms-21-03101-s001.zip › Supplemental Figures/Figure S2. Melting curves of miRNAs..tif]
